# Genital schistosomiasis in non-endemic settings: a clinical perspective

**DOI:** 10.1017/S0031182025100759

**Published:** 2025-12

**Authors:** Hannah Rafferty, Clare E. Warrell, Laura E. Nabarro, Gauri Godbole, Peter Chiodini

**Affiliations:** 1 Hospital for Tropical Diseases, London, UK; 2 Clinical Research Department, London School of Hygiene and Tropical Medicine, London, UK; 3 Rare and Imported Pathogens Laboratory, Porton Down, UK; 4 Malaria Reference Laboratory, London School of Hygiene and Tropical Medicine, London, UK

**Keywords:** clinical perspective, genital schistosomiasis, non-endemic settings

## Abstract

Genital schistosomiasis, caused mainly by infection with *Schistosoma haematobium* flukes, causes a variety of symptoms and significant complications in men and women. With high levels of migration from sub-Saharan Africa to Europe and North America, genital schistosomiasis is likely to be encountered more frequently by clinicians in non-endemic areas. In this article, we review the current knowledge of genital schistosomiasis in non-endemic areas, available guidelines and barriers to clinical care of patients. Future work to address these barriers will likely improve care for patients with this neglected and stigmatized disease.

## Graphical abstract

**Figure:**
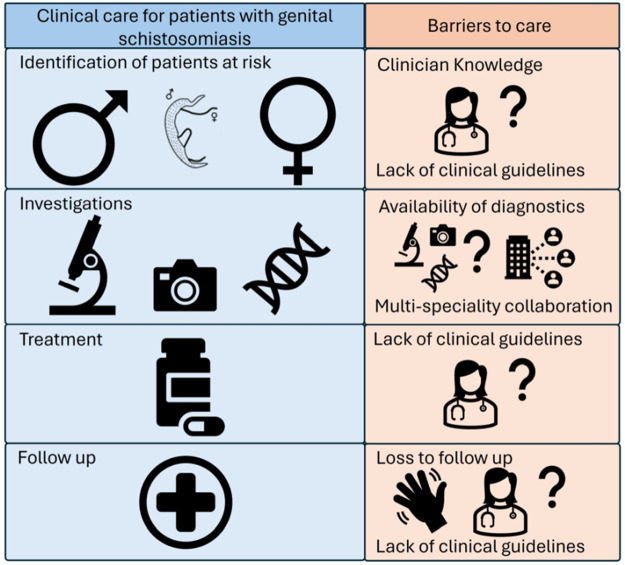

## Introduction

Genital schistosomiasis can affect men, in the form of male genital schistosomiasis (MGS), and women in the form of female genital schistosomiasis (FGS). It is an inflammatory condition, most commonly caused by infection with *Schistosoma haematobium* worms. *Schistosoma haematobium* worms form mating pairs in the urogenital venous plexus, producing eggs which migrate through genital tissue, causing inflammation in the reproductive organs (Bustinduy et al., 2022). This results in symptoms mimicking sexually transmitted infections and reproductive complications for affected men and women (Kjetland et al., 2010, 2012; Christinet et al., 2016; Laroche et al., 2016; Miller-Fellows et al., 2017; Kayuni et al., 2019). FGS is estimated to affect 56 million women, mainly in sub-Saharan Africa (Engels et al., 2020). Estimates are lacking for MGS, but this is likely to affect millions of men in the same region. With high levels of migration from sub-Saharan Africa to areas non-endemic for schistosomiasis such as Europe and North America, clinicians in non-endemic areas may more frequently encounter genital schistosomiasis (McAuliffe and Oucho, 2024). We review current knowledge and guidelines for clinical practice in non-endemic areas and explore potential barriers to care in these settings.

## Importance of diagnosis and management

Symptoms of FGS include abnormal bleeding, dyspareunia, genital lesions and abnormal discharge. Symptoms of MGS include lumpy semen, haematospermia, pelvic pain and pain during ejaculation. These symptoms can be mistaken for sexually transmitted infections, potentially leading to misdiagnosis and unnecessary treatments. A key complication of FGS is increased risk of HIV acquisition (Patel et al., 2021). Although further studies are required, it is hypothesized that MGS causes increased HIV-1 shedding in the semen. Left untreated, patients may develop complications, including subfertility in both men and women (Kini et al., 2009; Kjetland et al., 2010; Miller-Fellows et al., 2017; Bowers et al., 2025). FGS complications may also include ectopic pregnancy and stillbirth (Laroche et al., 2016). Given that schistosomiasis is a urogenital disease, genital schistosomiasis patients often also have urological manifestations, with a risk of squamous cell carcinoma of the bladder (IARC, 1994). In addition, there may be a link to cervical dysplasia (Rafferty et al., 2021; Sturt et al., 2025), and possibly prostate cancer (Figueiredo et al., 2015), although further research is required. Given the combination of genital symptoms, misdiagnosis of sexually transmitted infections and effects on fertility, genital schistosomiasis can be stigmatizing for many patients, and this may prevent patients from presenting to healthcare services. It is essential that genital schistosomiasis is diagnosed and managed promptly to avoid the host of potential associated complications and prolonged stigmatization of patients.

## Burden in non-endemic settings

There remain few studies estimating the burden of FGS in non-endemic settings, although FGS has been reported in both migrants and travellers from endemic areas (Chen et al., 2012; Laroche et al., 2016; Roure et al., 2022, 2024b). There have been two cross-sectional studies based in Barcelona including migrants from sub-Saharan Africa. These studies found schistosomiasis seroprevalence rates of 28.4% (Roure et al., 2024b) and 54.8% (Roure et al., 2022), and a strong association between seropositivity and gynaecological symptoms, including dysmenorrhoea, menorrhagia, vaginal discharge and pruritus and infertility. In a retrospective case notes review of patients presenting to the Hospital for Tropical Diseases in London with *Schistosoma* ova on microscopy of histopathology, only 6.5% of women reported gynaecological symptoms, highlighting potential diagnosis gaps (Rafferty et al., 2025). Further studies are urgently required in additional settings to assess the prevalence and impact of FGS in non-endemic settings.

Data on the burden of MGS in non-endemic settings are lacking, despite multiple reports of travellers and migrants presenting with MGS in non-endemic settings (Pérignon et al., 2007; Kini et al., 2009; Hawary et al., 2012; Richter et al., 2025). There is one community-based cross-sectional study of adult male migrants from sub-Saharan Africa living in Spain. This study reported a seroprevalence of 37.6%. Seropositivity was strongly associated with multiple genital symptoms, including pelvic pain, pain on ejaculation, erectile dysfunction and infertility (Roure et al., 2024a). In a recent retrospective case notes review of patients presenting to the Hospital for Tropical Diseases, 4.8% of male patients reported erectile dysfunction, and 6.5% self-reported infertility (Warrell et al., 2024). The full burden of MGS in non-endemic settings is yet to be established, with further studies required.

## Clinical guidelines for genital schistosomiasis

Clinical guidelines are helpful tools to aid diagnosis and management. Guidelines are especially useful for presentations that are rarely encountered by individual clinicians. Guidelines for schistosomiasis in endemic areas often follow WHO recommendations, which may be more focused on presumptive treatment and mass drug administration for schistosomiasis control (World Health Organization, 2022). Therefore, guidelines for the management of genital schistosomiasis specifically may be limited. The original FGS guideline was the WHO Atlas, suggesting FGS diagnosis via colposcopy and treatment with a single dose of praziquantel (Mbabazi et al., 2015). Further endemic guidelines include the COUNTDOWN FGS intervention manual, developed to assist primary healthcare workers in the diagnosis and treatment of FGS in Liberia (Nganda et al., 2023). The guideline prompts clinicians to ask about FGS symptoms, with a symptom and risk factor scoring system to indicate the need for colposcopy for diagnosis, followed by treatment if any visual signs of FGS are present. In addition, a screening tool has been developed in Zambia to help identify women at risk of FGS (Rogers et al., 2023). This includes using factors such as childhood exposure to lakes and streams, haematuria and occupation to guide treatment.

A literature search was conducted using PubMed and Google Scholar, searching for ‘genital schistosomiasis’, ‘guideline/s’ and ‘non-endemic’. This search identified three guidelines or clinical pathways available for FGS in non-endemic settings. The Italian consensus guideline includes a short section on FGS, suggesting colposcopy for diagnosis in at risk women (Comelli et al., 2023). The Spanish consensus guideline also briefly includes FGS, suggesting a diagnosis of FGS should be considered for women presenting with urogenital symptoms who have had contact with freshwater in schistosomiasis-endemic countries. These guidelines also suggest that absence of ova in the urine does not preclude a diagnosis of FGS, diagnosis is based on characteristic lesions at colposcopy (Bocanegra et al., 2023). Our group recently published an FGS clinical pathway for non-endemic settings based on results from a retrospective case notes review (Rafferty et al., 2025). This suggests asking all women with positive *Schistosoma* diagnostics about potential FGS symptoms and, if reported, referring to gynaecology for colposcopy for diagnosis, in addition to referrals to sexual health teams and urology to explore differential diagnoses and potential complications. No FGS guidelines were identified from North America or Oceania. All guidelines recommend a single dose of 40 mg kg^−1^ praziquantel for treatment of schistosomiasis regardless of genital manifestations.

During our search as detailed earlier, we identified no specific MGS guidelines for endemic or non-endemic settings. Consensus guidelines are urgently required to improve care of men with MGS, ensuring diagnosis, treatment and follow-up pathways that can be adapted to different clinical settings.

## Barriers to care for genital schistosomiasis

### Clinician knowledge

Lack of clinician knowledge of genital schistosomiasis is a major barrier to diagnosis. A cross-sectional survey for healthcare workers across Europe found that 56.3% of 581 doctors and 88% of 341 nurses surveyed were not aware of FGS (Marchese et al., 2025). Over two-thirds of doctors reported no knowledge, despite the majority of respondents working in infection or gynaecology specialties. Fewer than 10% of respondents worked in sexual health, where many FGS patients may present due to the symptomology. There have been no surveys examining knowledge of MGS in non-endemic settings. Given the relative lack of MGS research and clinical guidelines, clinician knowledge of MGS is likely to be poorer than FGS. Clinician awareness and knowledge of genital schistosomiasis are critical to improving patient care. Without understanding of symptoms, clinicians are unlikely to explore the full history with patients to identify those at risk of genital schistosomiasis, especially in specialties such as infection, where genital symptoms are not commonly scrutinized. Without direct questioning, patients may not volunteer these symptoms due to stigma or lack of awareness of relevance. This is evidenced in the Barcelona cross-sectional study, whereby direct questioning about genital symptoms via a questionnaire led to a significant increase in reported genital symptoms in women with schistosomiasis compared to standard clinical review (Roure et al., 2024). Importantly, patients with genital schistosomiasis may present to non-infection specialities, including primary care, sexual health, gynaecology and urology. If these specialties have poor knowledge of genital schistosomiasis, they will be unable to identify those requiring schistosomiasis testing and referral to infection specialties for further investigation and management. To improve knowledge and awareness, education initiatives are essential. These must include teaching for non-infection specialities to increase awareness of genital schistosomiasis and highlight at-risk individuals who would benefit from testing, and the management and follow-up of such patients. In addition, training must begin at medical school, with genital schistosomiasis included on the curriculum to ensure all clinicians have some knowledge and will be able to identify patients at risk and arrange appropriate diagnosis and management steps. In recent years, more interest has been paid to specific migrant health interventions, and genital schistosomiasis could form an important component of migrant health training in the future.

Given the overall lack of knowledge of genital schistosomiasis in non-endemic areas, understanding and consistency between individual clinicians of the investigations and management of patients with genital schistosomiasis is likely to be poor. Guidelines are an important measure for improving care for those identified as at risk for genital schistosomiasis. Given the potential complications of genital schistosomiasis and the additional risk of bladder carcinoma with this urogenital disease, investigation, treatment and follow-up must be robust. As described earlier in this manuscript, three guidelines or clinical pathways specifically for non-endemic settings are available, although these may need to be tailored to each healthcare setting depending on availability of diagnostics, speciality referrals and imaging capacity (Bocanegra et al., 2023; Comelli et al., 2023; Rafferty et al., 2025). This represents an additional challenge, as a ‘one size fits all’ approach may not be suitable given the complexities of referrals and investigations required, necessitating a further process of adaptation of guidelines and pathways for each centre.

To facilitate following of guidelines, clinicians require specific skills for the diagnosis of FGS. This includes the diagnosis of FGS via colposcopy. In 2024, the first edition of an international colposcopy course for the diagnosis of FGS- and HPV-related lesions was held. This course was open to clinicians from endemic and non-endemic areas. There are currently no available courses for clinicians in non-endemic settings to learn about colposcopy for FGS.

### Genital schistosomiasis diagnostics

The accuracy of schistosomiasis diagnostics has long been a barrier to the diagnosis and treatment of patients, given imperfect sensitivity and specificity (Vaillant et al., 2024). In the UK, the Hospital for Tropical Diseases recommends a combination of S*chistosoma* serology and microscopy of urine and stool for the diagnosis of schistosomiasis, followed by imaging of specific organ systems such as ultrasound of the urinary system and colposcopy for the diagnosis of FGS. In the UK’s National Health Service, schistosomiasis serology is usually a reference laboratory test, performed in London or Liverpool, which has associated costs. Urine and stool microscopy rely on experienced biomedical scientists, and with low incidence rates in non-endemic areas, scientists may become deskilled in this essential diagnostic. Circulating anodic antigen is a useful marker of active disease but can only be performed at Leiden University Medical Centre, the Netherlands. *Schistosoma*-specific PCR is an emerging diagnostic, mainly used in research settings, and is available for clinical specimens via a limited number of non-endemic centres. Although PCR may represent a sensitive and specific diagnostic tool, it cannot be used to monitor treatment efficacy as DNA may be detectable even after curative treatment. In one study 24% of women with FGS were persistently PCR positive 6 months after treatment (Downs et al., 2013). Follow up beyond 6 months for FGS, has not been completed, and there have been no post-treatment follow up studies for MGS, so it is not currently clear how long women or men might remain PCR positive following treatment. In addition, genital lesions may persist following successful treatment and clearance of infection, and so a negative PCR does not exclude potential complications of genital schistosomiasis.

Colposcopy is currently the recommended diagnostic for FGS; however, PCR on genital samples has been validated in various studies and in many FGS studies is used as the new ‘gold standard’ for diagnosis (Kjetland et al., 2009; van Bergen et al., 2024). Colposcopy can be subjective, relying on clinicians’ experience of FGS signs, although computer-aided diagnostic algorithms adapted from cervical cancer diagnostics are currently being explored to aid the objective diagnosis of FGS (Jin et al., 2023). Other than symptomology, MGS has no agreed diagnostics. PCR of semen has been successful in diagnosing MGS and is under further investigation (Kayuni et al., 2023). However, given very few centres offer PCR for clinical samples, these research advances are unlikely to be feasible for more widely accessible clinical use. The combination of imperfect accuracy of available investigations, with access and cost of each diagnostic, proves a challenge to diagnosis, even in high-resource settings.

### Multi-speciality collaboration

The investigation and management of genital schistosomiasis requires involvement of multiple specialties, given presenting symptoms, suggested investigations and differential diagnoses. This represents a challenge as each speciality individually needs to be aware of genital schistosomiasis, its presenting symptoms, diagnosis, management and follow-up. In addition, each speciality must agree to clinical guidelines requiring their input and approve proposed patient pathways. This becomes more challenging the more additional teams are involved. The management of FGS requires involvement of at least four different specialities: infectious diseases, sexual health, gynaecology and urology to fully investigate FGS, differential diagnoses and possible complications. Each speciality must approve the guideline and patient pathway before it can be implemented, which can be a challenge to coordinate. This also represents a barrier to a universal guideline, as the approach will need to be tailored for each involved speciality in each region, depending on wait times and available diagnostics.

In addition, schistosomiasis should be included in guidelines for other specialities. Currently, schistosomiasis is mentioned as a potential cause of epididymo-orchitis in the British Association for Sexual Health and HIV guideline (Chirwa et al., 2021), although no further information about relevant history and investigation is provided. The National Institute of Clinical Excellence (NICE) fertility guideline does not include schistosomiasis as a potential cause of subfertility (O’Flynn, 2014). Ideally, these guidelines would be updated to highlight patients at risk of genital schistosomiasis and how to investigate.

### Barriers to care for migrants

Migrants from sub-Saharan Africa are at the highest risk of genital schistosomiasis. Migrants face numerous barriers to accessing healthcare, which also need to be considered and addressed where possible when creating clinical pathways. Barriers include health literacy and knowledge of the health system in host country, language differences, documentation and legal challenges, financial barriers and unstable housing situations with need to relocate frequently, amongst many others (Lebano et al., 2020; Nowak et al., 2022). These factors affect the initial presentation to services, and retainment in follow-up. Loss to follow up is likely to be a key issue with genital schistosomiasis, given the number of different investigations and speciality reviews patients may need, and the long-term follow-up for complications that may be required. Ideally, pathways would be integrated into other services such as cervical cancer screening, sexual health screening and holistic migrant health services. In addition, transition of care between healthcare providers may be required if patients relocate during follow-up.

### Barriers to care preventing patient presentation to healthcare

**Figure 1.**
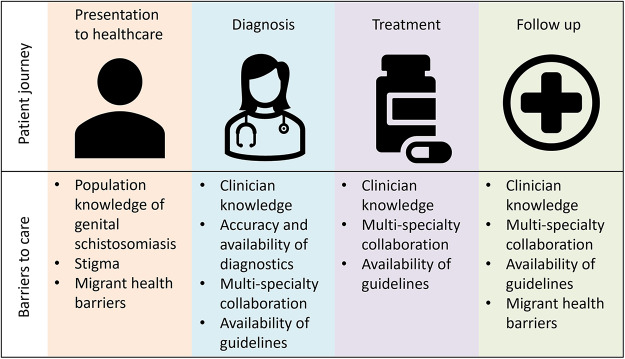
Schematic of patient journey for genital schistosomiasis, with associated proposed barriers to care.

Along with the above-described barriers to care for migrants, multiple other barriers exist for all patients that may prevent initial presentation to healthcare. Symptoms may be mild, or ongoing for a prolonged duration and so normalized for individual patients, preventing them from seeking care. In addition, certain symptoms may be culturally normalized and so not identified as an issue requiring intervention. To combat these barriers, targeted population health education may be helpful to improve public knowledge of genital schistosomiasis and its symptoms and importantly inform patients that effective treatment exists and can prevent future complications. Stigma due to genital symptoms and complications may prevent patients from seeking healthcare and can be a prominent feature of genital schistosomiasis. Peer support from people with known genital schistosomiasis amongst high-risk populations may encourage healthcare-seeking behaviours and reduce stigma. Gender differences in healthcare-seeking behaviour are well documented, with men routinely having fewer healthcare consultations than women (Bertakis et al., 2000; Wang et al., 2013; Ballering et al., 2023). This may lead to underreporting of MGS and the burden of disease in men. Perhaps with improved public knowledge and peer support specifically for MGS, more men would be encouraged to present to healthcare services. It is essential to build trust and rapport between populations at risk of genital schistosomiasis and healthcare services. Successful treatment and compassionate care from clinical teams will help, alongside community education and peer support programmes. All barriers to care for genital shcistosomiasis are summarised in figure 1.

## Conclusions

Genital schistosomiasis is likely to be more commonly encountered in non-endemic settings with high levels of migration from sub-Saharan Africa. Improved clinician knowledge of genital schistosomiasis is essential, and genital schistosomiasis should be included in medical school curricula and through to relevant speciality training, including practical courses for skill acquisition. Given potential complications, comprehensive diagnosis, management and follow-up pathways are essential. This can represent a challenge given the lack of clinician knowledge, variability in diagnostics and multiple specialities coordinating patient pathways. Furthermore, these pathways will need to be adapted to each centre, depending on service availability. Progress has been made with published FGS clinical pathways for non-endemic settings; however, MGS is lagging behind, with no clinical guidelines yet published for endemic or non-endemic settings. Further research into genital schistosomiasis in non-endemic settings is urgently needed to fully understand the burden of disease and ensure robust patient pathways to improve the care of patients with this neglected disease.
